# To Be FAIR: Theory Specification Needs an Update

**DOI:** 10.1177/17456916251401850

**Published:** 2026-01-22

**Authors:** Caspar J. Van Lissa, Aaron Peikert, Maximilian S. Ernst, Noah N. N. van Dongen, Felix D. Schönbrodt, Andreas M. Brandmaier

**Affiliations:** 1 Department of Methodology and Statistics, Tilburg University; 2 Center for Lifespan Psychology, Max Planck Institute for Human Development, Berlin, Germany; 3 Max Planck UCL Centre for Computational Psychiatry and Ageing Research, Berlin, Germany; 4 Max Planck School of Cognition, Leipzig, Germany; 5 Faculty of Law, University of Amsterdam; 6 Department of Psychology, Ludwig-Maximilians-Universität München; 7 Department of Psychology, MSB Medical School Berlin

**Keywords:** fairtheory, metascience, theory formation, open science

## Abstract

Open science innovations have focused on rigorous theory testing, yet methods for specifying, sharing, and iteratively improving theories remain underdeveloped. To address this limitation, we introduce *FAIR theory*, a standard for specifying theories as findable, accessible, interoperable, and reusable digital objects. FAIR theories are findable in well-established archives; accessible in terms of their availability and ability to be understood; interoperable for specific purposes, such as selecting control variables; and reusable in that they can be iteratively and collaboratively improved on. This article adapts the FAIR principles for theory; reflects on current FAIR practices in relation to psychological theory; and discusses FAIR theories’ potential impact in terms of reducing research waste, enabling metaresearch on theories’ structure and development, and incorporating theory into reproducible research workflows—from hypothesis generation to simulation studies. We present a conceptual workflow for FAIRifying theory that builds on existing open science principles and infrastructures. More detailed tutorials, worked examples, and convenience functions to automate this workflow are available in the *theorytools* R package. FAIR theory constitutes a structured protocol for archiving, communicating about, and iteratively improving theory, addressing a critical gap in open scholarly practices and potentially increasing the efficiency of cumulative knowledge acquisition in psychology and beyond.

The FAIR Guiding Principles (hereafter: FAIR principles) were established by a diverse consortium of stakeholders to improve the reusability of research data and other resources produced in the course of scholarly work by making them findable, accessible, interoperable, and reusable (M. D. Wilkinson et al., 2016). Since the FAIR principles’ inception, they have become a widely adopted standard for archiving academic output, representing an estimated tens of billions of dollars in reuse value (Vogt et al., 2024). Scholars have demonstrated their relevance for making other digital objects more open, including research software (Lamprecht et al., 2019) and computational workflows (Van Lissa, Brandmaier, et al., 2021; S. R. Wilkinson et al., 2024). In the current article we argue that the FAIR principles can similarly advance effective and transparent scholarly communication about theory. We introduce *FAIR theory*, a digital instantiation of scientific theory published as a self-contained and citable digital object distinct from—but potentially associated with—the scientific article. Definitions of theory abound and are hotly debated, but because many are compatible with the FAIR principles, this article is not limited to one particular definition. FAIR theory can potentially improve the transparency and efficiency of scholarly communication, reduce research waste, and accelerate cumulative knowledge acquisition. We focus on applications in psychology, but the principles are relevant across the social sciences and beyond.

## The Need for FAIR Theory

The so-called replication crisis has prompted extensive scientific reforms (Lavelle, 2021; Scheel, 2022). Concern that the abundance of nonreplicable findings was caused by undisclosed flexibility in analyses led to the widespread adoption of open science practices such as preregistration and replication (Nosek et al., 2015). These various practices ensure transparent and repeated testing of hypotheses by committing to an analysis plan in advance. However, recent reviews have shown that most preregistered hypothesis tests are not supported by empirical evidence (Scheel, Schijen, & Lakens, 2021).

Increased rigor in testing has revealed that the root cause of the replication crisis is more fundamental: Psychological theories rarely provide hypotheses that are corroborated by evidence. Furthermore, theories are often so ambiguous that they can accommodate mutually inconsistent findings, rendering them immune to falsification. Consider the self-determination theory (SDT; Deci & Ryan, 2012), one of the most widely cited social psychological theories that we formalized in this vignette (see https://cjvanlissa.github.io/theorytools/articles/formalizing_sdt.html). The SDT emphasizes the role of intrinsic and extrinsic motivation in human behavior. Intrinsic motivation was initially defined as engaging in an activity purely for the inherent satisfaction it provides, free from any external rewards or pressures (Deci, 1971). Over time, however, this definition expanded to include motivations driven by the fulfillment of basic psychological needs for autonomy, competence, and relatedness (Ryan & Deci, 2000). The implications of these shifting definitions become clear when deriving hypotheses about the type of motivation involved in, for example, changing an infant’s dirty diaper. Under the original definition, one would hypothesize that caregivers are not intrinsically motivated to change diapers because this is hardly a joyous experience. Under the expanded definition, one would hypothesize the opposite because the act fulfills the need for relatedness. Expanding the definition thus enables the SDT to absorb potentially falsifying evidence.

Scholars have raised concerns about the state of theory in psychology for nearly 50 years (Meehl, 1978; Robinaugh et al., 2021). One frequently raised concern is that theories lack formalization (Szollosi & Donkin, 2021). When theories are ambiguous, precise predictions cannot be derived from them without resorting to subjective interpretation or invoking additional assumptions, which makes them harder to falsify. A second concern that has received less attention is the lack of transparent and participative scholarly communication about psychological theory, which limits its progression and development. Despite these concerns, scientific reform initiated by the open science movement has focused primarily on improving deductive methods. The equally critical processes of theory construction and improvement have been largely overlooked. The current article addresses this knowledge gap by applying, for the first time, open science principles to psychological theory. We introduce FAIR theory as a methodology that can facilitate transparent scholarly communication and accelerate cumulative knowledge acquisition.

## What Is Theory?

Given that a pluriformity of definitions are consistent with FAIR theory principles, we do not limit ourselves to any one particular definition—although our writing inevitably reveals a particular vantage point. Perspectives on scientific theory have been categorized as syntactic, semantic, and pragmatic (Winther, 2021). The syntactic view describes theories as “*sets of sentences in a given logical domain language*” (Winther, 2021, Section 2, para. 1), acknowledging that each domain (a scientific field such as psychology or physics) has its own theoretical vocabulary. We recognize the syntactic view in Meehl’s (1990) hierarchy of evermore specific “statements” a theory might contain: statements about the types of entities postulated (i.e., ontology, Level 1), statements about causal connections between those entities (Level 2), statements about the functional form of those connections (Levels 3–8), and statements about their specific numerical values (Level 9). The semantic view challenges the necessity of distinct domain languages for different scientific fields and instead advocates for formalizing theories using mathematics. It shifts the focus from theories as collections of sentences to mathematical models. The term “model” is not uniquely defined within the literature; models have been described as “specific instantiations of theories, narrower in scope and often more concrete, commonly applied to a particular aspect of a given theory” (Fried, 2020, p. 336). This implies that theories and models are not fundamentally distinct but rather, for each model, there is a more general theory that subsumes it (one person’s model is another person’s theory). The pragmatic view holds that there might not be one structure or definition of scientific theories and instead that definitions differ across scientific domains. It also holds that nonformal aspects (e.g., commonly used analogies) and practices (e.g., experimental designs) can be an important part of scientific theories.

## Theory and Scientific Progress

**Figure 1.**
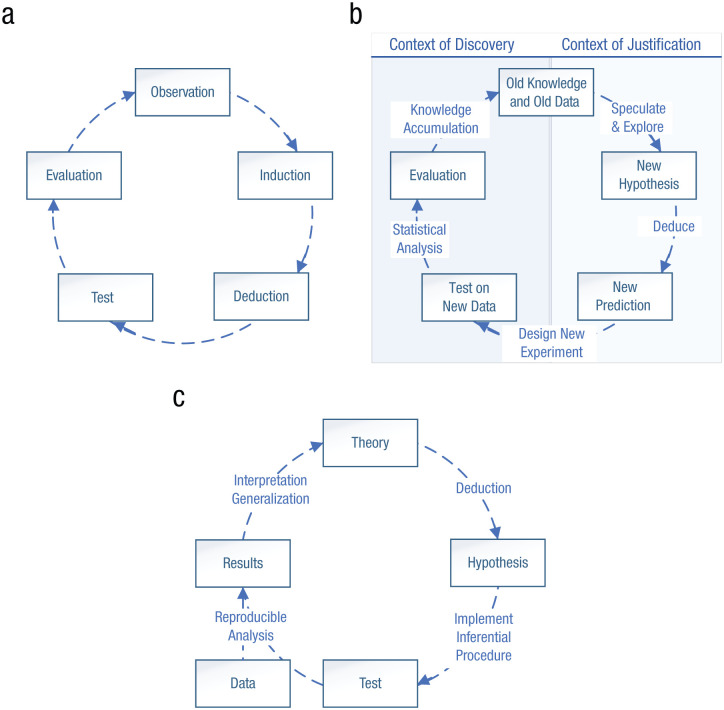
Three implementations of the “empirical cycle.” Implementations of the empirical cycle are shown from (a) De Groot & Spiekerman (1969), (b) Wagenmakers et al. (2018), and (c) Van Lissa (this paper, see the section “Changing a theory”).

According to the *empirical cycle* (De Groot & Spiekerman, 1969), a metatheory of cumulative knowledge acquisition, research ideally follows a cyclical process (Fig. 1a). Naturalistic observations or patterns identified in data give rise to preliminary hypotheses via induction. Deduction is then used to derive predictions from these hypotheses, which are tested empirically using data. Last, the outcomes of tests are evaluated with regard to their implications for theory. Wagenmakers et al. (2018) divided this cycle into two phases (Fig. 1b). One phase is the “context of justification,” in which hypotheses derived from theory are tested on data. The other phase is the “context of discovery,” in which patterns observed in data are generalized to theoretical principles. In these interpretations of the empirical cycle, each stage describes a process. This invites one to consider the concrete outcomes of these processes. For example, how does knowledge accumulate when iterating through the cycle? What actually changes, other than the scholar’s mind? To address these questions, the first author (C. J. Van Lissa) specified a revised version of the empirical cycle (see Changing a Theory section) in which the nodes refer to specific deliverables and the edges describe processes acting on those deliverables (Fig. 1c). In this specification, “theory” refers to FAIR theory, “hypothesis” refers to machine-readable hypotheses (Lakens & DeBruine, 2021), “test” refers to a preregistered inferential procedure (Preregistration as Code; Peikert et al., 2021), “data” refers to FAIR data, and “results” refer to (preprint) manuscripts, ideally supplemented with a comprehensive reproducible research archive (Van Lissa, Brandmaier, et al., 2021). In this model, theories are the vehicle of scientists’ understanding of phenomena; they are what changes when iterating through the cycle.

In a progressive research program (Lakatos, 1971), this cycle is regularly completed to iteratively advance our understanding of the studied phenomena via deductive testing and inductive theory construction. There are, however, indications that contemporary psychology falls short of this ideal. Meehl observed that theories in psychology “lack the cumulative character of scientific knowledge. They tend neither to be refuted nor corroborated, but instead merely fade away as people lose interest” (Meehl, 1978, p. 1). Recent empirical findings confirm this view: Hypothesis-testing research is vastly overrepresented in the literature, amounting to 89.6% of published articles (Kühberger et al., 2014). A closer examination of such studies reveals, however, that the link between theory and hypothesis is often tenuous or absent (Oberauer & Lewandowsky, 2019; Scheel, Tiokhin, et al., 2021). Only 15% of hypothesis-testing studies referenced any theory, and rarely in direct relation to the hypothesis (McPhetres et al., 2021). Theory thus has an uncomfortable and paradoxical role in contemporary psychology; that is, although most research ostensibly tests hypotheses, these hypotheses are rarely connected to theory.

Perhaps some ungrounded hypotheses are rooted in implicit theories privy only to the author, in which case it would be useful to make them explicit (Fried, 2020; Norouzi et al., 2024). Or perhaps some hypotheses are not of substantive interest but merely reported as part of entrenched cultural practices (Gigerenzer et al., 2004), such as straw-man null hypotheses that exist solely for the purpose of being rejected (Van Lissa, Gu, et al., 2021). Testing ad hoc hypotheses not grounded in theory, or grounded in misinterpreted or multi-interpretable theory, cannot advance our principled understanding of psychological phenomena and consequently contributes to research waste (Nakagawa et al., 2024). Collecting significance statements about ad hoc hypotheses is much like trying to write novels by collecting sentences from randomly generated letter strings (van Rooij & Baggio, 2021)—inefficient at best and, more likely, futile. Because the Declaration of Helsinki prescribes that ethical (medical) research with human participants must “avoid research waste,” our field should take seriously its ethical responsibility to develop procedures to reduce it. The current article does so by introducing procedures to improve transparent, unambiguous, and efficient communication about theory and thus instantiating theory as a digital “object” that scholars can access, reuse, and update in their daily workflows.

## Making Theory FAIR

Merely publishing theory in a journal article does not make it open; to be open, theory should adhere to established open science standards for specification and archival. We propose implementing theories as digital objects and archiving these digital objects with appropriate metadata in a FAIR-compliant repository such as Zenodo, which was developed by CERN under the European Union’s OpenAIRE program (European Organization For Nuclear Research & OpenAIRE, 2013). Metadata are “data about the data.” They provide information about the nature and content of a digital object and are stored in the repository in which the version of record of the FAIR theory is deposited. FAIR theories are findable via a digital object identifier (DOI) or by searching the repository in which they are archived; accessible in a machine- and human-readable filetype; interoperable for specific purposes, for example, within the data-analysis environment; and reusable in the practical and legal sense so that they may be iteratively improved on by the author or by others. Following the original proposal of Lamprecht et al. (2019), we adapted the FAIR principles for theory (see Table S1 in the Supplemental Material available online). We reflected on the necessary (minor) changes as well as on the current state and future of FAIR theory in psychology. The resulting principles provide guidance for instantiating theory as a FAIR digital object, and we provide worked examples to encourage their adoption.

### Findability

Making theories findable would allow researchers to easily identify relevant theories and ground their hypotheses in established theoretical foundations. It could increase the impact and reuse potential of theories across disciplines, either through direct application (in which one discipline stumbles on a problem that is already well understood in another discipline) or through analogical modeling. In analogical modeling, the structure of a theory from one discipline is applied to a phenomenon in another field. For example, predator–prey models have inspired theories of intelligence (Van Der Maas et al., 2006), and the Eysenck model of atomic magnetism has inspired a network theory of depression (Cramer et al., 2016). Findability also enables metaresearch on theories in the same way libraries and search engines have enabled scholars to study the literature via systematic reviews. In a similar way, it would become much easier to explicitly compare different theories of a specific phenomenon, or to study structural properties of theories.

The four findability criteria are applicable to theory with only minor adjustments (see Table S1). First, a globally unique and persistent identifier, such as a DOI, should be assigned to each theory (see criterion F1 in Table S1). Of the many services that provide DOIs for archived objects, Zenodo and OSF are commonly used in psychology.

Second, findable theory is described with rich metadata (F2). Using standardized metadata further improves the findability of digital objects. The DataCite Metadata Schema provides a controlled vocabulary for research output (DataCite Metadata Working Group, 2024). For example, data are typically archived with the metadata property resourceType: dataset. The resource_type: model would be more appropriate for archiving FAIR theory. If data were collected for a specific article, that relationship could be cross-referenced with relationType: IsSupplementTo. Similarly, a FAIR theory can be connected to a theory article using relationType: IsDescribedBy, whereas the reverse relationship, documented in the theory article, is relationType: Describes. Other cross-references are useful for documenting relationships between multiple theory objects: If an existing theory is made FAIR without substantial alterations, the resulting FAIR theory metadata would cross-reference the existing theory as relationType: IsDerivedFrom. If an existing theory is updated, relationType:IsNewVersionOf could be used to reference previous versions. If a variation of an existing FAIR theory is created, cross-reference it with relationType: IsVariantFormOf. Other relevant metadata might be a reference to a taxonomy of psychological constructs (Bosco et al., 2017), ontology (Guyon et al., 2018), or catalogue of psychological phenomena. Metadata should also include identifiers for all versions of the theory it describes (F3); Zenodo handles this by default by providing an overarching DOI for a digital object that subsumes the DOIs of its subversions.

Last, metadata should be registered or indexed in a searchable registry (F4). We propose using the keyword “fairtheory,” as we do in the current article, for all resources that constitute or reference (a specific) FAIR theory so that they can easily be found. It is important to note that, although many archives are technically searchable (e.g., GitHub, FigShare, OSF, institutional repositories), only few are specifically designed for FAIR-compliant archival. Zenodo stands out in this respect.

The findability of a resource is substantially amplified if its intended users know where to search for it. This is a known problem in relation to research data and software (Katz & Chue Hong, 2024). Regrettably, most academic search engines index only traditional print publications, not other digital objects. Thus, one reason to continue publishing theory articles alongside FAIR theories is to meet the findability criterion by publishing a theory article in print, using the DataCite Metadata Schema to connect both resources. The “fairtheory” keyword can also be used in print publications to signal their association with a FAIR theory. In the longer term, it may not be necessary to write an article for each theory. If Zenodo gains recognition as a centralized repository for digital objects, researchers may begin to search there more regularly. Conversely, as organizations (e.g., Google Scholar, Web of Science, Pure, ORCID) begin to recognize other forms of academic output beyond articles, they may begin to index digital objects stored in Zenodo.

There have been notable efforts to improve theories’ findability through post hoc curation. For example, Gray and colleagues introduced a format for representing theories and posted many examples on their website (see https://www.theorymaps.org; Gray, 2017). Similarly, PsychoModels (see http://psychomodels.org/) seeks to inventory theories and models in psychology. Post hoc curation is a notable effort but does not address the root cause of the lack of findability. Ideally, findability would be addressed ante hoc through documentation with rich metadata and modular publishing. Both approaches can be complementary, however. For example, post hoc curation could make use of existing FAIR-compliant archival infrastructure such as Zenodo. The data-engineering adage “lots of copies keeps stuff safe” implies that it is fine to archive theories in multiple places, although it is advisable to make use of automatic integration (as exists between the GitHub (http://github.com/), Zenodo (https://zenodo.org), and OSF (https://osf.io) platforms) to avoid the need to maintain information in multiple places, which increases the risk of inconsistencies arising.

### Accessibility

Accessibility in scholarly communication about theory implies that researchers and other stakeholders (e.g., practitioners, policymakers, advocates) can inform themselves of the current scientific understanding of specific phenomena. If theories are not accessible, researchers cannot reuse and refine them, which impedes cumulative knowledge acquisition. Similarly, stakeholders cannot use inaccessible theories to make evidence-based decisions, which curtails the societal impact of scholarly knowledge. Accessible theories are also an important instrument in science communication: Whereas isolated empirical findings can appear fragmented and contradictory (Dumas-Mallet et al., 2017), theories offer a top-down, big-picture representation of the phenomena studied in a field.

The accessibility principles apply to theory with minor changes. First, theory and its associated metadata should be accessible by their identifier using a standardized communications protocol (A1). This can be achieved, for example, by hosting theory in a version-controlled remote repository from which it can be downloaded via an application programming interface. Such platforms include GitHub, which is well suited for collaborative theory development, and Zenodo, which is better suited for long-term storage of the version of record. The resulting resource will have an identifier (DOI) that allows it to be accessed using a standardized communications protocol (download via https or git). Second, theory metadata should be accessible even when the theory is no longer available (A2). In general, it makes sense to retain outdated theories to be able to track their genesis over time. The A2 criteria imply that—even when theories are removed or lost for whatever reason—they should be archived on a platform on which the theory’s metadata are indelible. Archival infrastructure such as Zenodo meets this need.

At present, there are several impediments to theories’ accessibility. First, when theories are published in paywalled journal articles, they are in principle accessible to paying readers but not practically to all. Open-access publishing increases the practical accessibility of all academic output, including theory. A second, more indirect impediment to theories’ accessibility is their intelligibility. It has been proposed that good theories have the property of “discursive survival . . . the ability to be understood” (Guest, 2024, p. 1). At present, psychological theories are often ambiguous, rendering them difficult to understand (Frankenhuis et al., 2023). Successful communication requires shared background knowledge between sender and receiver (Vogt et al., 2024). This knowledge can come from shared paradigms (Kuhn, 1996), from education, and from the available methods and instrumentation—or it can be problematically absent. Accessibility is improved by explicitly referring to sources of assumed background knowledge and by reducing unnecessary ambiguity. At the same time, it is important to acknowledge that it is impossible to remove all ambiguity when communicating an idea. The *indeterminacy of translation* holds that every communicative utterance (e.g., a statement in natural language, a mathematical formula) has multiple alternative translations, with no objective means of choosing the correct one (Quine, 1970). This places a theoretical upper bound on theories’ ability to be understood.

A third impediment arises when theories have a “dependency on the author” (DOA). A DOA occurs when a theory cannot be understood by independent scholars, requiring the original author to provide interpretation and clarification. DOAs relate to the discourse on “great man theorizing” (Guest, 2024) because they enable gatekeeping: Authors could insist that work requires their involvement or denounce work conducted outside their purview as illegitimate, which violates the checks and balances of scientific research. DOAs also render theories immune to refutation because authors can claim that their theory was misconstrued when confronted with falsifying evidence, thus making it a moving target (Szollosi & Donkin, 2021). DOAs are inherently problematic, as illustrated by cases in which third parties identify logical inconsistencies within a theory (e.g., Kissner, 2008). This example demonstrates that original authors are not the ultimate authority on their theories. DOAs thus unduly impede scientific progress.

In sum, authors should make good-faith efforts to make theories as accessible as possible in terms of availability, intelligibility, and freedom from dependencies that cannot be resolved (including DOAs or manuscripts that can no longer be accessed with reasonable effort). Although the indeterminacy of translation places an upper bound on interpretability, scholars should nevertheless strive to reduce unnecessary ambiguity to the greatest possible extent. It may benefit scientific discourse to normalize explicit ambiguity (i.e., things we don’t know yet) and anticipate misunderstanding to invite others to fill in the blanks and motivate ever further explication of theory. A theory’s accessibility is increased by reducing dependencies on (implicit) background knowledge, explication of assumptions, formalization, and explicit cross-references to relevant resources such as articles, ontologies, and other related theories, measurement instruments, and experimental designs (Lange et al., 2025).

### Interoperability

Interoperability pertains to the property of digital objects to “integrate or work together . . . with minimal effort” (M. D. Wilkinson et al., 2016, p. 2). To be interoperable, theories and associated metadata should use a formal, accessible, shared, and broadly applicable language to facilitate (human and) machine readability and reuse (I1). The common practice of instantiating theory as lengthy prose or schematic drawing falls short of this ideal. Instead, FAIR theory should, at a minimum, be specified using a human- and machine-readable data type, as are other digital objects resulting from scholarly work (e.g., data, analysis code, software; Van Lissa, Brandmaier, et al., 2021). Depending on the theory’s level of formalization, different formats may be appropriate, such as verbal statements in plain text, mathematical formulas, or statements expressed in some formal language (e.g., pseudocode, interpretable computer code, Gray’s theory maps; Gray, 2017).

Second, theory (meta)data should use vocabularies that follow FAIR principles (I2). Aside from the aforementioned DataCite Metadata schema (DataCite Metadata Working Group, 2024), in the context of theory, this highlights the importance of establishing standardized ontologies. Third, theory (meta)data should include qualified references to other (meta)data, including previous versions of the theory (I3). The first part of this principle allows for nested theories; for example, a theory that specifies causal relationships between constructs could refer back to an ontological theory from which those constructs are derived. This can be achieved by cross-referencing the DOI of those nested theories (DataCite, 2024). The second part of this principle allows for tracing the provenance of a theory—keeping track of its prior versions and other theories that inspired it. This is achieved by using Git for version control and Zenodo for archiving. Git tracks the internal provenance of a theory repository; Zenodo is used to cross-reference external relationships (e.g., articles that influenced the theory, previous theories that inspired it, models based on the theory).

Recent work has pointed out that interoperability is not an all-or-nothing property. The concept of “X-interoperability” was introduced to answer the question “Interoperable for what?” X-interoperability is defined as facilitating “successful communication between machines and between humans and machines . . . [where] A and B are considered X-Interoperable if a common operation X exists that can be applied to both” (Vogt et al., 2024, p. 5). This revised definition makes it possible to outline a theory’s affordances in terms of X-interoperability. For a practical example, consider the empirical cycles in Figure 1. If these are instantiated as an image (bitmap or raster graphic), then they are X-interoperable only for reproduction in print—and even then they cannot be resized or edited without a loss of quality. If the same theories are instantiated in the DOT graph description language (Graphviz, 2024), then they can be displayed, resized, and edited before being printed without a loss of quality. Graphs in the DOT language can additionally be edited at the conceptual level, which is a convenient property for theories that are expected to be updated over time, and they can be loaded into statistical programming software such as R and converted to causal graphs or into a rudimentary generative model for simulation studies. Thus, the way a theory is instantiated affects its X-interoperability.

X-interoperability is also affected by the type of information encoded in a theory. If we consider Meehl’s (1990) nine levels of strong theories (p. 114), we see how each of these levels enables specific types of interoperability. Level 1, specifying an ontology, enables X-interoperability for selecting relevant variables; Level 2, causal connections, enables deriving testable predictions about associations, covariate selection, and causal inference; and Levels 3 through 8 refer to functional form, which allows for deriving specific hypotheses about unknown model parameter values and specifying statistical models for estimating them. If Level 9 is also specified, and parameters are assigned specific numerical values, the theory is completely specified; it is a digital twin of the system it seeks to describe and can be used to evaluate hypothetical scenarios and construct generative models to produce synthetic data via simulation.

With regard to the state of interoperability in psychology, Lewin’s (1943) adage “there’s nothing as practical as a good theory” paints a hopeful picture of theories as useful tools in psychological researchers’ daily workflows. But, as we have argued, contemporary practice falls short of this ideal. Much can be gained by integrating theory directly into analysis workflows and by making theory X-interoperable within analysis software. For example, interoperable theory can be used to select control variables for causal inference (Cinelli et al., 2022), or to preregister a study with an explicit derivation chain from theory to hypothesis, as well as an inferential procedure that would suggest specific modifications to theory after analyzing empirical data (Peikert et al., 2021), or to derive machine-readable hypotheses (Lakens & DeBruine, 2021) that could be automatically evaluated through integration testing (Van Lissa, 2023). Furthermore, theories can be X-interoperable with each other to enable nesting, or using one theory to clarify elements of another theory. For example, it should be possible to embed a theory about emotion regulation (e.g., Gross, 2015) within a theory of emotion regulation development (Morris et al., 2007).

### Reusability

If we take cumulative knowledge acquisition to be a goal of scientific research, then reusability is the ultimate purpose of making theory FAIR. Applied to FAIR theory, reusability requires that each theory and its associated metadata are richly described with a plurality of accurate and relevant attributes (R1) with a clear and accessible license for reuse (R1.1). It should further have detailed provenance (R1.2), which is achieved through version control with Git and archival on Zenodo. Last, the (meta)data should meet domain-relevant community standards (R1.3). The DataCite Metadata schema offers an initial template in this regard, and this article takes one step toward establishing more fine-grained community standards for FAIR theory. This (see https://github.com/cjvanlissa/fair_theory/blob/3b4894da576cb76d19e911a05dd513d5172058ec/example_metadata.json) is an example of FAIR metadata extracted from Zenodo.

If we consider the current state of reusability in psychological theory, there appears to be a norm against theory reuse: “[Theories are] like toothbrushes — no self-respecting person wants to use anyone else’s” (Mischel, 2008, p. 1). Because cumulative knowledge acquisition requires reusable theories that are continuously updated on the basis of insights from new data, such a norm impedes scientific progress (De Groot & Spiekerman, 1969). In FAIR theory workshops, we similarly notice a reluctance to reuse and adapt existing theories. Students ask questions such as “Who owns a theory?” and “Who determines how a theory may be reused or changed?” These questions imply a norm against modifying theory without its author’s consent, reminiscent of the aforementioned DOA problem.

Licensing theories for reuse unambiguously answers these questions, with the caveats that legislation may vary across contexts and jurisdictions and that the current article does not constitute legal advice. Two considerations are important when determining what license is appropriate for theory. A first consideration is that copyright law protects authors’ rights according to the idea-expression dichotomy (Bently et al., 2010). Copyright does not “extend to any idea, procedure, process, system, method of operation, concept, principle, or discovery” (Copyright Act, 2024, Section 102b). Copyright thus applies to creative works expressing a theory (e.g., prose, visual illustrations) but not to the underlying theoretical idea. It thus seems that theories expressed in prose or depicted visually—in other words that fall short of the accessibility criterion—are more likely to qualify for copyright protection than formal theories. A second consideration is that academic research is covered under “fair use” exemptions to copyright. Given these two considerations—that copyright does not protect ideas in their purest form and that academic use offers exemptions to copyright—it may be counterproductive and possibly misleading to adopt a license that assumes copyright protection to theories. For psychological theories without commercial aspects, we suggest using a license that explicitly waives copyright and encourages reusability, such as CC0 (no rights reserved).

Aside from legal conditions for reuse, there are also social considerations. For example, although a CC0 license does not legally mandate attribution, the norms of good scientific practice mandate that scholars comprehensively cite theory and related works (Aalbersberg et al., 2018). Particularly when FAIRifying an existing theory, failing to credit its author amounts to scientific malpractice. Another instrument for guiding the social process of (diffuse) collaboration is to include a README file in the theory repository that informs users about the ways in which they can reuse and contribute to a FAIR theory. A final suggestion is to create or adopt a “code of conduct” that prescribes behavioral norms for contributors and users of a theory, for example, the Berlin Code of Conduct (https://berlincodeofconduct.org/en).

## Relevant Considerations

### What to archive?

As FAIR theory becomes more commonplace, we expect that it will become increasingly clear what kind of information is useful and how theory and models should be archived. As a particular FAIR theory develops, details may be added, and the nature of the information tracked might even change. For example, following Meehl, we could envision a theory that starts out with establishing an ontology of relevant constructs through observation of a given phenomenon. After initial exploratory research, the theory might be further specified by making assumptions about how these constructs are causally connected. Over time, more precise *statistical/mathematical* models could be derived by further assuming a specific functional form for relationships (e.g., linear effects) and error families for the distribution of measured variables (e.g., normal distributions). This would allow for the specification of statistical models, which make just enough assumptions to allow for the estimation of the remaining unknown parameters (e.g., regression slopes) from data. Going even further, a *generative/computational* model could be specified that is completely parametrized (e.g., specific values of regression slopes are also assumed) such that an interpreter (e.g., the R programming language) can use the model to generate new data. Aspects of scientific practice might also be added over time—either to the theory itself or as references recorded in the theory metadata. Examples include experimental designs (e.g., longitudinal designs observing change over time), measurement instruments (e.g., different questionnaires used to assess the same construct), or information about participant recruitment and retention strategies.

Theories can include or reference other theories (nesting). For example, consider a comprehensive theory of disease spread and pandemics that covers various psychological factors such as adherence to infection prevention protocols (e.g., social distancing), pandemic-related behavior (e.g., panic buying), and pandemic-related distress (Taylor, 2022). This theory of disease spread may encompass another FAIR theory that specifies a mathematical model of disease transmission, with precise parameters for the process of infection (e.g., social distance, average duration of encounters, ventilation) and incubation times. Metadata can be used to properly cross-reference the relationship between the larger theory of disease spread and the transmission model it subsumes.

### The role of theory formalization

Concerns about the state of psychological theory have motivated increasing calls for greater theory “formalization” (Smaldino, 2017; cf. Oude Maatman, 2021). Formalization increases theories’ falsifiability (Popper, 2002) because it expresses ideas as specific statements, clearly demarcating what should (not) be observed if the theory were true. Take, for example, the phonological loop in Baddeley’s (1992) working memory model. The idea behind it is that verbal information is kept in memory via a rehearsal loop with limited capacity. The verbal description of the phonological loop in Baddeley’s theory of working memory stands out for clarity and comprehensibility, yet it allows for at least 144 different implementations depending on the specification of various parameters such as decay rate of information, recall success, or rehearsal sequence, which were left undefined in the original theory (Lewandowsky & Farrell, 2010). Without committing to specific implementations a priori, the theory becomes hard to test. Compared with theories expressed in natural language, formal theories facilitate inconsistency checking and evaluation of a theory’s (lack of) vagueness.

Committing to specific implementations makes a theory more precise, and precise theories are easier to falsify. If a theory is static, as is the case for print publications, falsifying evidence may cause it to become irrelevant. There is thus a perverse incentive to propose theories that are hard to refute because these will persist in the scientific record unchallenged. FAIR theory offers an alternative approach, in which theory can be falsified without losing relevance because it can be continuously updated. Researchers can commit to very precise implementations, allowing for tests that would necessitate specific revisions in the face of falsifying evidence, thereby advancing our principled understanding of phenomena.

Crucially for the current article, FAIR theory imposes no restrictions on the manner in which theories are derived and implemented; rather, it pertains to the rigorous and transparent archival and communication about theories, with the aim of enhancing their reusability. FAIR theory does not require formalization, and formal theories are not automatically FAIR. In other words, formalization is orthogonal to FAIRification. The FAIR principles apply equally to theories represented in natural language and in formal theories represented using mathematical notation, algorithmic pseudocode, or a set of logical clauses. Thus, for example, “grounded theory,” derived from qualitative research, can be represented as a FAIR theory using plain-text propositions. Conversely, a formal theory is not FAIR if it is confined to a journal article without any key words to identify it as a theory article (lacking findability), represented merely as a bitmap image (limiting accessibility and interoperability), or subject to copyright (limiting reusability). FAIR theory is thus consistent with, but does not require, formalization (see also Accessibility section below). This principle is illustrated in our vignette on FAIRifying De Groot’s empirical cycle (see https://cjvanlissa.github.io/theorytools/articles/fair-theory.html): It is equally possible to FAIRify the theory in its original formulation by archiving a text document with five plain-language propositions or to formalize the theory and represent it as a human- and machine-readable diagram before FAIRifying it.

### Modular publishing

The primary unit of scientific communication has long been the academic article. Yet scholars often produce many other valuable resources in the process of writing articles, including instruments, materials, data, code, and theory. These resources are often merely described in articles and not made available for reuse. Modular publishing is the practice of making each of these resources available as independent digital objects, facilitating their reuse and making them citable (Van De Sompel et al., 2004). At the time of writing, some modular publishing practices are already widely adopted; data sharing, for example, has become the de facto standard in psychology in the past decade (Tedersoo et al., 2021). We envision FAIR theory as another instance of modular publishing (Kircz, 1998).

Modular publishing can be achieved by archiving specific resources (including theory) in FAIR-compliant repositories such as Zenodo. Modular publishing increases resources’ reuse potential and makes them citable. This does not detract from the conventional academic article as a unit of academic communication with more room for nuance and the author’s voice. Theories published in traditional articles can be supplemented by FAIR versions that live independently, evolve collaboratively, and feed into reproducible workflows.

### Version control

The field of computer science provides inspiration for well-established processes for iteratively improving digital objects. Version control systems, such as Git, are used to iteratively improve computer code while managing parallel contributions from collaborators and allowing for experimentation and diverging development without losing information. Git tracks line-by-line changes to text-based files and maintains a complete annotated history of those changes. It has previously been argued that Git is particularly well suited to academic work (Ram, 2013; Van Lissa, Brandmaier, et al., 2021). For example, Git can facilitate reproducible research, manage distributed collaboration, and improve preregistrations (Peikert et al., 2021; Van Lissa, Brandmaier, et al., 2021). Git provides a useful framework for developing FAIR theory because it enables explicitly comparing versions of a file (or theory), documenting why changes were made, incorporating changes by different authors, and branching off into different directions (e.g., competing hypotheses) while retaining an explicit link to the common ancestor. This makes it possible for metascientists to study the provenance of a theory and determine how well different versions of a theory explain empirical evidence (Van Lissa, 2023). Cloud platforms associated with Git (e.g., GitHub) facilitate collaborative theory development, for example, by making it possible to duplicate an entire theory archive and develop it in a new direction (technical term: *forking*), suggesting changes to an existing theory archive (*pull request*), and explicitly comparing differences between versions of a theory (*comparing commits*). However, cloud platforms such as GitHub are less suitable for archiving the version of record because of a lack of FAIR compliance. Thus, theory development may take place on GitHub, but versions of record should be archived on a platform such as Zenodo, with appropriate metadata.

### Semantic versioning

Aside from technical solutions, version control is a social process as well. On one hand, regular updates can improve theories; on the other hand, however, it risks breaking compatibility between theories and hypotheses derived from them, or compatibility between one theory and others that depend on it. For example, if we construct a theory to explain a specific phenomenon and we cross-reference an existing theory comprising an ontology for our field, that dependency is broken if the ontology is later updated and our phenomenon of interest is removed. In computer science, these challenges are navigated by assigning version numbers. Specifically, *semantic versioning* comprises a simple set of rules for assigning version numbers to digital objects. Whereas version control tracks changes, semantic versioning communicates what those changes mean to users of the theory, guides the social process of theory development, and signals how much a theory has been changed.

We propose adapting semantic versioning for theories by assigning a version number in the format MAJOR.MINOR.PATCH (e.g., 0.1.0), where the MAJOR number is incremented when backward-incompatible changes are made, for example, if the theory now contains empirical statements that are at odds with a previous version of the theory. The MINOR number should be incremented when the set of empirical statements are expanded in a backward-compatible manner (i.e., the previous version is subsumed within the new version). The PATCH number should be incremented when making backward-compatible bug fixes and cosmetic changes, fixing spelling errors, or adding clarifications.

## FAIR Theory Workflow

We present a conceptual workflow for making theory FAIR to give readers some sense of the steps involved. Although these steps can be implemented using a variety of tools, the *theorytools* R package automates most steps. This package includes a worked example (see https://cjvanlissa.github.io/theorytools/articles/fair-theory.html) for implementing this workflow that, as a living document, can be kept up to date with changing infrastructures. The package further includes tutorial examples for FAIR theory creation based on existing substantive theories, including an example of how to formalize and FAIRify the SDT (see https://cjvanlissa.github.io/theorytools/articles/formalizing_sdt.html; Ryan & Deci, 2000), how to FAIRify Morris’s tripartite model of parental socialization of children’s emotions (see https://cjvanlissa.github.io/theorytools/articles/causal-inference.html; Morris et al., 2007) and use it for causal inference, and how to FAIRify a mathematical model based on the Dunning-Kruger effect (see https://cjvanlissa.github.io/theorytools/articles/dunning-kruger.html; Feld et al., 2017).

To prevent the emergence of an open science “cottage industry,” we recommend using existing open science infrastructures to the greatest possible extent. The integration of GitHub and Zenodo currently makes for a particularly user-friendly approach that meets all FAIR principles. Zenodo and GitHub are both integrated with OSF, a popular platform in psychology. Thus, it is possible to create a project page on OSF to increase the visibility of a FAIR theory among users of that platform, whereas the integration of OSF with Zenodo and GitHub removes the need for maintaining the same information on multiple platforms. Note that open science infrastructure is an area of active development and that workflows might change as new tools or databases are developed or existing tools and databases change over time.

1. Implement the theory

Imagine that one would want to FAIRify De Groot’s empirical cycle, a metatheory of theory construction. Begin by creating an empty folder to hold all files associated with the theory—this folder will become the theory archive. The first file to create is the theory itself. This could be a plain-text file containing natural language statements, or a more formal representation, such as a directed graph. For example, the empirical cycle was originally described as a series of natural language statements (De Groot & Spiekerman, 1969):

Phase 1: ‘Observation’: collection and grouping of empirical materials; (tentative) formation of hypotheses.

Phase 2: ‘Induction’: formulation of hypotheses.

Phase 3: ‘Deduction’: derivation of specific consequences from the hypotheses, in the form of testable predictions.

Phase 4: ‘Testing’: of the hypotheses against new empirical materials, by way of checking whether or not the predictions are fulfilled.

Phase 5: ‘Evaluation’: of the outcome of the testing procedure with respect to the hypotheses or theories stated, as well as with a view to subsequent, continued or related, investigations. (p. 28)

Implementing the theory as a digital object can be as simple as saving these statements to a plain-text file.

Optionally, we can formalize the theory further. According to a taxonomy of different levels of theory formalization (Guest & Martin, 2021), the empirical cycle is currently defined at either the “theory” or “specification” level. To fulfill Criterion I1 of the FAIR principles using a formal language for knowledge representation (see Table S1), we can further formalize it to the “implementation” level by specifying it in the DOT language for describing directed graphs.^1^ Given the cyclical nature of the conceptual model, such an implementation might look like this:


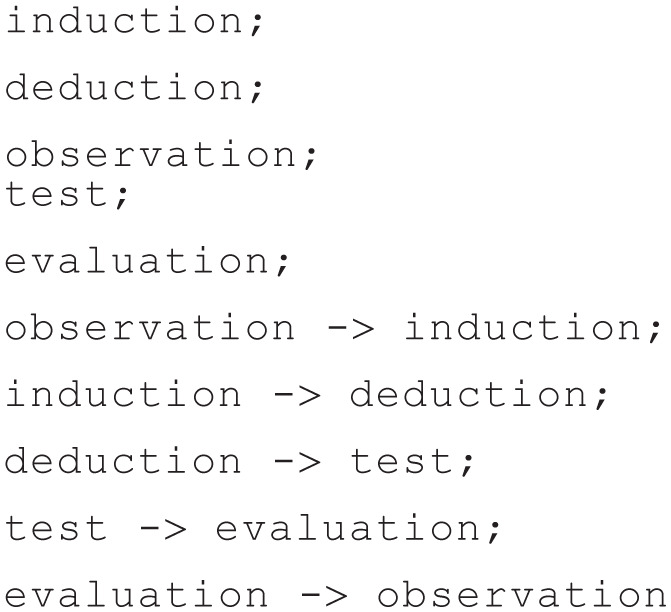


Note that the first part of the implementation constitutes an ontology—it specifies the entities contained in the theory. The second part of the implementation describes the flow of information from phase to phase. Fig. 1a shows what this implementation looks like when plotted. Regardless of which implementation we prefer, we can save it to a plain-text file—this is the “digital object” containing our theory.

2. Document the theory

To meet the interoperability and reusability criteria, it is important to properly document the theory file. First, add a README.md file with instructions for future users of your theory. The *theorytools* package contains a vignette on writing README files for theory (see https://cjvanlissa.github.io/theorytools/articles/readme.html. Second, add a LICENSE file with the legal conditions for reuse. We recommend explicitly waiving copyright with the CC0 license, but other options are available (see https://choosealicense.com).

3. Version control the theory archive

To track all changes to our theory, the theory archive can be version controlled. Git is well suited for this purpose. Hosting a backup in the cloud on a platform such as GitHub additionally makes the theory publicly accessible and facilitates community engagement.

4. Archive the theory on Zenodo

Archiving major versions of a theory in a FAIR-compliant repository that issues a persistent identifier (DOI) improves their findability and allows them to be referenced in perpetuity. Zenodo is a FAIR-compliant repository with GitHub and OSF integration.

5. Entering metadata

When archiving a FAIR theory, documenting it with relevant metadata improves its findability. We recommend using a standardized metadata schema such as DataCite (DataCite Metadata Working Group, 2024). Within this schema:

- Set the resource type to Model.
- Add the words “FAIR theory” to the title so that sentient readers will recognize the work as a FAIR theory (just as meta-analyses should use the words “meta-analysis” in the title).
- Add “fairtheory” to the keywords to aid search engine indexation (using “FAIR theory” causes some search engines to look for either the word “fair” or “theory,” which would be overly inclusive).
- Optionally, submit the theory to the FAIR Theory Community (see https://zenodo.org/communities/fairtheory) to contribute to community building; communities on Zenodo are shared spaces to manage and curate research outputs.

The FAIR implementation of De Groot’s empirical cycle that resulted from the first author (C. J. Van Lissa) implementing this workflow is available at https://doi.org/10.5281/zenodo.14552329.

### Changing a theory

An important advantage of FAIR theory is that we can implement different versions of a theory, compare them, and document their cross-relationships. We can take work that has been done before—in this case the repository created above—and create an independent copy that we can modify as we wish while retaining cross-references to the original. Elaborating on our running example, we can implement Wagenmakers et al.’s (2018) take on the empirical cycle (Fig. 1b). Their interpretation differed from De Groot’s in several ways: First, they referred to the phases by different names, and this change was not described in the article. Assuming that these new names were merely intended to be illustrative and not ontologically distinct, we could incorporate this change by adding labels to the original ontology. These labels would suggest a focus on empirical psychology that was not present in De Groot’s version. Wagenmaker et al. (2018) explicitly mentioned a second change: “We added the Whewell-Peirce-Reichenbach distinction between the context of discovery and the context of justification” (p. 423). We could implement this change to the original implementation by grouping the respective phases of the cycle—a minor and tractable change:


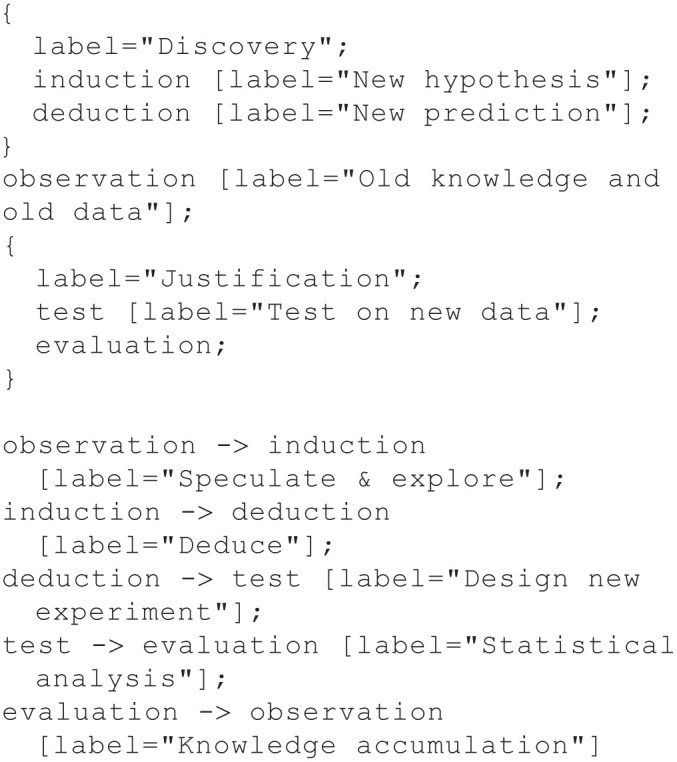


The current work is guided by an interpretation of the empirical cycle in which nodes refer to specific FAIR digital objects (FAIR theory, machine-readable hypotheses, FAIR data, etc.), and edges connecting the nodes refer to processes acting on (X operating with) those digital objects (Fig. 1c). This implementation addresses an important ambiguity of prior versions of the empirical cycle. De Groot described how the phase of evaluation involves the “formation of new [theories]”; evaluating results from one iteration of the cycle thus become observations (Phase 1) that initiate a new iteration. De Groot stopped short of detailing the process of theory construction or revision, and in Wagenmakers’s version, the term “knowledge accumulation” also remained unspecified. Thus, in both prior versions, it is unclear *how* knowledge accumulates in the evaluation phase. In Van Lissa’s (2025) specification, “theory” is understood as FAIR theory, which has clear procedures for making changes and connecting these changes to empirical evidence.

A second departure from De Groot is that the processes of induction and deduction are intentionally abandoned as distinct phases. Theory testing, as takes place in the “context of justification,” can be said to involve *mostly* deductive reasoning. Theory development and amendment, as takes place in the “context of discovery,” involves mostly inductive reasoning.^2^ However, deriving hypotheses from theory is not *entirely* deductive because auxiliary assumptions must often be made to account for remaining ambiguities in theory, which involves induction (Peikert, 2023). A common example is assuming equal variances across groups when testing a hypothesis about a mean difference between groups. One might justify this assumption because groups often have equal variances (induction from prior knowledge), or because a Levene’s test on the data set at hand is nonsignificant (induction from a specific observation to the population). Additionally, if we accept that observation is theory-laden, then it too involves induction (Brewer & Lambert, 2001). These alterations result in the following implementation of the empirical cycle:


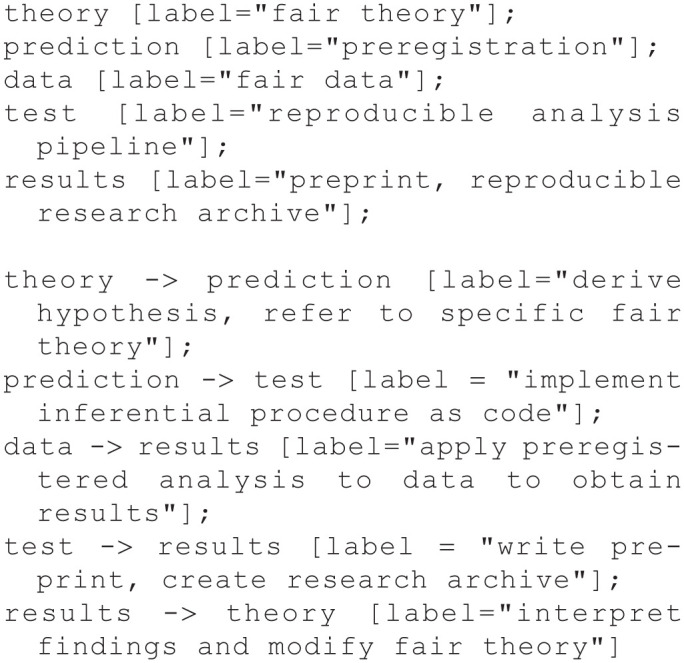


The FAIR theory workflow offers concrete ways to make changes to a FAIR theory object and to compare different versions, as explained here (see https://cjvanlissa.github.io/theorytools/articles/updating_theory.html).

### Further uses of FAIR theory

As uses of FAIR theory are best illustrated using tutorial examples, the *theorytools* package contains several vignettes that showcase specific applications. The package currently includes a vignette introducing augmented directed acyclic graphs (aDAGs; see https://cjvanlissa.github.io/theorytools/articles/augmented_dags.html) as a format for theory specification that meets the requirements of good psychological theory. These aDAGs are X-interoperable for plotting (using dagitty and tidySEM), for automatically selecting control variables, and for simulating data (using *theorytools*), as described in this vignette (see https://cjvanlissa.github.io/theorytools/articles/causal-inference.html; Pearl, 1995). Other vignettes may be added over time, and users are encouraged to submit their own reproducible examples as package vignettes.

## Discussion

The replication crisis has brought the inadequacies of contemporary theoretical practices in psychology and other fields into focus. Psychological theories often fall short of the FAIR principles: They are hard to find and access, have no practical uses in scholars’ daily workflows beyond providing context for a literature review, and are more likely to be forgotten or replaced than reused. These limitations impede cumulative knowledge production in our field, leading to an accumulation of “one-shot” empirical findings, without commensurate advancement in our principled understanding of psychological phenomena. We argued that applying the FAIR principles to theory offers a structured solution to these shortcomings. We demonstrated how to create, version control, and archive theories as digital objects. We introduced the *theorytools* R package to partly automate these processes, reducing the barrier of entry for researchers and creating a FAIR resource for theory-construction tools and documentation that can be updated as best practices continue to develop.

Making theory FAIR allows researchers to more easily find a relevant framework; access and understand it; interact with it in a practical sense, for example, by deriving predictions from it, or using it to select control variables; and reuse it, contributing changes to existing theories or splitting off in a new direction. Whereas “theory” can be a nebulous concept to empirical psychologists, FAIR theory makes it practical and tangible, incorporating theory into scholarly workflows. Having a concrete object to iterate on facilitates the systematic improvement and iterative refinement of psychological theories, thus substantially increasing the efficiency of research. Although FAIR theory does not directly reduce vagueness, it provides a framework within which scholars can iteratively increase precision and formalization. The FAIR principles also facilitate new ways of collaboration, leveraging tools such as Git for version control and Zenodo for archiving to document provenance and facilitate contributions from diverse researchers.

### How to incentivize FAIR theory development

FAIR theory requires a departure from contemporary practice. Several factors can expedite such a culture change. One key factor is the *recognition and rewards* movement: Practices for evaluating scientific output are evolving, with initiatives such as the Declaration on Research Assessment and the Coalition for Advancing Research Assessment promoting the use of more diverse and meaningful metrics beyond journal impact factors. Modular publishing capitalizes on these changing metrics, and FAIR theory allows scholars to be credited for theoretical contributions (Kircz, 1998). Journals that publish theoretical articles could require authors to additionally publish their theories in a FAIR format, cross-referencing the article to expedite its effective reuse and iterative improvement. A second factor is to lower barriers to adopting FAIR theory by building on existing widely adopted open science infrastructures. For this reason, we advocate the use of Git for version control, Zenodo for archiving, and DataCite for standardized metadata. Barriers to entry can also be lowered by simplifying workflows, which is the goal of the *theorytools* R package. Fourth, the availability of open educational materials about theory development contributes to doctoral socialization (Schönbrodt, 2025; N. van Dongen, 2025). These materials allow teachers to incorporate theory development into their curriculum without investing substantial time on course development, thus educating the next generation on how to make use of and contribute to FAIR theory. Last, community building plays an important role; the international network of open science communities, reproducibility networks, and other similar initiatives provide platforms for disseminating FAIR theories and related methodological innovations. Authors can also share their FAIR theories with other early adopters by submitting them to the FAIR Theory Community on Zenodo.

### Strengths

One important strength of FAIR theory is that it provides much needed open science methods for the underemphasized inductive phase of the empirical cycle. Recently, the open empirical cycle was introduced, positing that each phase in De Groot’s model of cumulative knowledge generation via scientific research can be supported by specific open science practices to increase the transparency, quality, trustworthiness, and replicability of research (Hoijtink et al., 2023). As we identified, however, most existing open science methods focus on rigor in testing (Phases 2–4 of the cycle), but few provide guidance on how to derive hypotheses from theory (Phase 1) or how to relate empirical findings back to theory (Phase 5), leaving a gap in the cycle. By instantiating theory as a FAIR digital object, we provide much needed open science infrastructure for transparently deriving hypotheses and modifying theory, thus contributing to closing the open empirical cycle.

Our approach aligns closely with recent developments in open science, such as modular publishing, interdisciplinarity, metaresearch, and team science. The advantage of modular publishing is that authors can be credited for theory development. Given the current emphasis on empirical articles (McPhetres et al., 2021), theoretical work can be hard to publish. FAIR theories, by contrast, can be readily disseminated as citable digital objects, thus changing the incentive structure to favor theory development. FAIR theory’s accessibility can advance interdisciplinarity, for example, because theoretical frameworks can be reused, adapted, or used for analogical modeling across different fields (Haslbeck et al., 2022). Metaresearch benefits from the fact that FAIR theory enables studying the structure, content, and development of theories over time. In terms of team science, FAIR theory facilitates collaboration by ensuring that all contributors have access to the same information and clarifying any remaining areas of contention or misunderstanding (Van Lissa et al., 2024). Version control provides a framework for resolving parallel developments from multiple collaborators in a nondestructive manner. This facilitates collaboration across geographical boundaries, and adversarial collaboration, in which others strive to falsify a theory or identify its inconsistencies. Version control also democratizes collaboration with as-of-yet unknown collaborators via platforms such as GitHub, where researchers outside one’s network can identify issues or suggest improvements to theories.

### Limitations

One important limitation of the current work is that, although it builds on well-established infrastructures such as Zenodo, it is unlikely that the proposed workflow is definitive. Community adoption can reveal areas of further improvement. Furthermore, dedicated indexing systems for FAIR theory are currently nonexistent. Using the Zenodo search function and submitting theories to the FAIR Theory Community on Zenodo can help overcome this limitation in the short term.

Another limitation is the learning curve associated with tools and infrastructures such as Git and Zenodo. The *theorytools* R package mitigates this limitation for R users by automating key steps in the process. Moreover, the initial investment in time can be offset by long-term productivity gains and increased impact of FAIR theory. One final way to address the learning curve is via specialization and collaboration, or team science (Van Lissa et al., 2024): As scientific workflows increase in sophistication, it is increasingly difficult for any one scholar to master all skills involved. In relation to FAIR theory, we see unique opportunities for intergenerational collaboration and knowledge exchange because theoreticians tend to be seasoned experts, whereas open science literacy is more commonly found among early-career scholars.

One potential barrier to adopting FAIR theory is cultural resistance to sharing and modifying theories (the “toothbrush problem”). Education might help address this limitation; with this in mind, we have shared several open educational materials on theory development in the FAIR Theory Community on Zenodo, and we encourage others to reuse these and share their materials.

A limitation of scope is that FAIR theory does not directly resolve problems related to strategic ambiguity (Frankenhuis et al., 2023) and lack of theory formalization (Robinaugh et al., 2021). However, our work does establish a framework that allows for and promotes the formalization of theories. The example of the empirical cycle demonstrates how FAIR principles can guide theory formalization and foster cumulative progress. Another limitation of scope is that FAIR theory does not resolve other related issues in psychology, such as the measurement crisis (Bringmann et al., 2022) and lack of standardized ontologies for psychological constructs (Bosco et al., 2017). In their seminal article on construct validity and the use of nomological nets, Cronbach and Meehl (1955) discussed crucial insights that are compatible with a FAIR theory approach. They described the interdependence between construct validity and the validity of theories that include that construct, proposing that the entire body of empirical evidence should be evaluated in relation to both (p. 4) and emphasizing that doing so requires theory specifying a “theory sufficiently clearly that others can accept or reject it” (p. 13). Our work here provides an infrastructure for realizing this vision. For example, if FAIR theories reference standardized ontologies and operationalizations for theoretical constructs, and if hypotheses’ machine-readable and inferential procedures are implemented using reproducible code, then it would become possible to evaluate the entire body of empirical evidence (FAIR data) against these machine-readable tests in an ongoing manner—similar to a continuously cumulating meta-analysis.

### Future directions

One important future direction is embedding FAIR theories withing existing open science methodologies. For example, consider how FAIR theory relates to preregistration. These practices are distinct but complementary. FAIR theory allows scholars to communicate general principles and expectations about a given phenomenon and to provide infrastructure for explicitly deriving hypotheses from specific theories and revising those theories in light of empirical results. Preregistration, by contrast, allows scholars to eliminate inductive bias from hypothesis tests and increase trust in the outcomes of a specific empirical study (Peikert et al., 2023). FAIR theories are specified at a level of abstraction that transcends individual studies. FAIR theories can inform—and be informed by—both quantitative and qualitative research. Preregistrations, by contrast, are typically specific implementations of quantitative hypothesis tests within the context of a specific study design, analysis plan, and—optionally—a fully reproducible analysis pipeline (although they are also used for other purposes, including for qualitative and exploratory research). These practices complement each other: Authors can make the derivation chain from theory to hypothesis more explicit by citing a specific FAIR theory in their preregistration. Moreover, it is possible to preregister an inferential procedure that would require revising the theory after observing data, or even to have proponents and detractors of a theory review a registered report of such a test. In short, combining FAIR theory with preregistration and other existing open science practices has the potential to strengthen the epistemic cycle of prediction, testing, and revision, moving us closer to a cumulative science.

Another future direction is the intersection between the aforementioned “theory crisis” and the related “measurement crisis” pertaining to the lack of clarity, consistency, and validity in the operationalization of theoretical constructs (Bringmann et al., 2022). FAIR theories could reference specific measurement instruments, or even theories of measurement, when operationalizing constructs named in a theory. FAIR theories can also help address “jingle-jangle fallacies” in psychology, that is, ambiguities that arise from using the same term for different constructs or, conversely, using different terms for the same construct (Song et al., 2021). By explicitly referencing operational definitions in FAIR theories, such fallacies would come to light and could ultimately be resolved.

Another future direction for FAIR theory is as an instrument of science communication. Practitioners and the general public are rarely able to read and derive actionable insight from large quantities of empirical articles about a particular phenomenon. Theories are more accessible because they encapsulate the bigger picture of contemporary scientific understanding. For example, although few people read empirical studies on attachment, attachment theory plays a prominent role in popular scientific books about parenting and romantic relationships. Theory bridges the gap between academic research and practitioners by summarizing actionable insights, relieving practitioners from the need to sift through extensive empirical literature. By providing a mechanism for iterative improvement based on emerging evidence, FAIR theory also supports effective evidence-based decision-making.

Last, although this article has discussed the potential impact of FAIR theory in addressing contemporary challenges in psychology, future research is needed to evaluate whether this potential is realized. Registered reports were first introduced to psychology about a decade ago (Nosek & Lakens, 2014) and are now becoming commonplace, as are studies evaluating their adoption and impact on scientific practices and research results (Scheel, Schijen, & Lakens, 2021). We envision a similar trajectory for FAIR theory.

## Conclusion

How would adopting a FAIR framework for theory construction, improvement, and reuse affect scholarly workflows? FAIR theory can be used to derive and justify hypotheses, thus addressing the problem that most hypotheses in psychology are currently atheoretical (McPhetres et al., 2021). Archiving theories in a FAIR manner also clarifies the version of record and makes changes traceable. This can be useful in preventing DOAs, distinguishing a theory from the predictions it makes, tracking how empirical findings are used to motivate changes to the theory, and resolving theoretical disputes. Although we have argued that FAIR theory performs some functions more effectively than traditional theory articles, it does not necessarily replace them altogether. The important question is what the unique contribution of a theory article will be if FAIR theories become commonplace. The Taskforce Theoretical Psychology developed a template for theory articles that addresses this question (Van Lissa et al., 2025). Importantly, FAIR theory can evolve beyond an original print publication, offering a persistent and collaborative object that others can reuse, cite, and refine—thus allowing scholars to more efficiently complete the empirical cycle. Developing FAIR theory could be a new type of scholarly output and should be recognized and rewarded as such according to the Coalition for Advancing Research Assessment.

We envision a future in which applying the FAIR principles makes theories more useful, living up to Kurt Lewin’s ideal, enabling scholars to incorporate theory into their workflows in a tangible way, providing explicit derivation chains for hypotheses, applying transparent rules for selecting the right control variables for causal inference, and proposing specific changes to existing theories on the basis of empirical results. FAIR theory is a major step forward toward more transparent, collaborative, and efficient theory construction. It provides much needed open science methods for the inductive phase of the empirical cycle, closing a critical gap in the scientific process. This paves the way for more theory-driven scholarship and accelerates cumulative knowledge acquisition in psychology, the social sciences, and beyond.

## Supplemental Material

sj-csv-1-pps-10.1177_17456916251401850 – Supplemental material for To Be FAIR: Theory Specification Needs an Update Supplemental material, sj-csv-1-pps-10.1177_17456916251401850 for To Be FAIR: Theory Specification Needs an Update by Caspar J. Van Lissa, Aaron Peikert, Maximilian S. Ernst, Noah N. N. van Dongen, Felix D. Schönbrodt and Andreas M. Brandmaier in Perspectives on Psychological Science

## Footnotes

1. Presented here in a simplified form; see the tutorial here (https://cjvanlissa.github.io/theorytools/articles/fair-theory.html) for technical details.

2. Here, “induction” is defined as forming general theories on the basis of specific observations. Others have used the term “abduction” to describe “inference to the best explanation” (Peirce, 1960). For the current purposes, the terms are interchangeable.

## Transparency

*Action Editor:* Scott Vrieze

*Editor:* Arturo E. Hernandez

*Author Contributions*

**Caspar J. Van Lissa:** Conceptualization; Formal analysis; Funding acquisition; Methodology; Project administration; Software; Supervision; Writing – original draft; Writing – review & editing.

**Aaron Peikert:** Conceptualization; Formal analysis; Writing – original draft; Writing – review & editing.

**Maximilian S. Ernst:** Writing – review & editing; Investigation.

**Noah N. N. van Dongen:** Writing – review & editing.

**Felix D. Schönbrodt:** Conceptualization; Writing – review & editing.

**Andreas M. Brandmaier:** Formal Analysis; Writing – original draft; Writing – review & editing.

**Open Practices:** This article is reproducible given the code provided on GitHub and can be accessed using Commit ID d4662e41.
